# Combined therapy with intravitreal aflibercept and subtenon corticosteroids in eyes with severe diabetic papillopathy: two case reports

**DOI:** 10.1186/s13256-021-03129-1

**Published:** 2021-10-22

**Authors:** Ilir Arapi, Piergiorgio Neri, Alfonso Giovannini, Arjeta Grezda

**Affiliations:** 1 The Eye Clinic, Experimental Sciences, Polytechnic University of Marche, Via Conca, 71, Torrette, AN 60126 Italy; 2 Eye Insitute, Cleveland Clinic Abu Dhabi, Abu Dhabi, United Arab Emirates; 3 The Eye Clinic, Mother Theresa University Hospital, Tirana, Albania

**Keywords:** Diabetic papillopathy, Aflibercept, Subtenon triamcinolone acetonide

## Abstract

**Background:**

Diabetic papillopathy is a rare diagnosis of exclusion characterized by unilateral or bilateral optic disc edema with variable degrees of visual loss. Although the visual prognosis has been generally reported as favorable, the presence of severe disc edema associated with macular edema prompts the need for treatment. We present a specific and unreported therapeutic approach consisting of intravitreal aflibercept and subtenon triamcinolone acetonide injections in two patients with evidence of diabetic papillopathy and macular edema.

**Case presentation:**

In the first case, a 60-year-old Caucasian woman affected by type II diabetes mellitus presented with fundoscopic evidence of sequential bilateral optic disc edema associated with acute severe visual loss in both eyes. The second patient, a diabetic 57-year-old Caucasian male, presented with sudden painless visual loss in his left eye. Multimodal imaging and systemic findings correlated towards an infrequent diagnosis of diabetic papillopathy. In a period of 5–7 weeks after treatment, both patients experienced almost full visual and anatomical recovery. A steady situation was observed at 12 months of follow-up.

**Conclusions:**

Both our cases displayed a severe grade of optic disc edema, which was optimally reversed with intravitreal aflibercept and subtenon triamcinolone acetonide leading to a relatively rapid and safe improvement in visual acuity.

## Background

Diabetic patients are susceptible to an uncommon form of optic neuropathy that is characterized by an acute optic disc edema with a relative absence of significant optic nerve dysfunction [1]. Diabetic papillopathy (DP) seems not to be associated with the severity of diabetic retinopathy (DR), while it affects one or both eyes and has been described in either type I or type II diabetes [2, 3]. Although the pathophysiology of DP remains to be fully elucidated, a combination of factors focused on the optic disc such as diabetic microangiopathy and a small disc have been reported as possible mechanisms [1, 4, 5]. Owing to the lack of a specific treatment protocol, different approaches were described, employing corticosteroids and anti-vascular endothelial growth factor (VEGF) injections, in order to reduce the disc swelling.

To the best of our knowledge, herein we report on the first successful employment of a combination therapy including subtenon triamcinolone acetonide injection (STTAI) and intravitreal aflibercept injection (IAI) in two patients who were affected by DP.

## Case reports

### Case 1

**Fig. 1:**
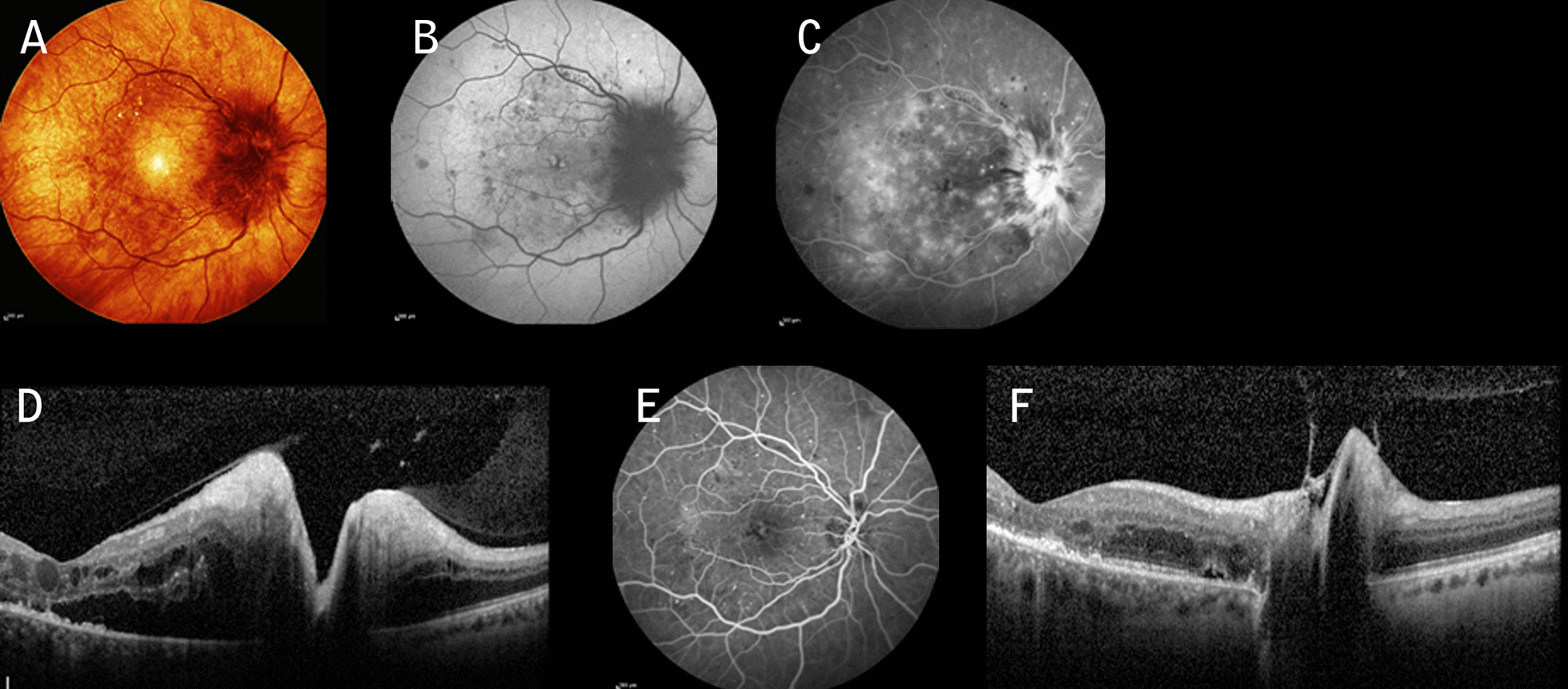
**A** Infrared image showing severe swelling of the nerve (left eye), splinter hemorrhages, and superficial dilated vessels associated with nonproliferative diabetic retinopathy. **B** Fundus autofluorescence showing diffuse peripapillary hypoautofluorescence and foveal hyperautofluorescence spots consistent with macular edema. **C** Fundus fluorescein angiography highlighting hyperfluorescence due to telangiectatic capillaries during the late venous phase. **D** Spectral domain optical coherent tomography revealing a significant increase in retinal nerve fiber layer thickness, serous retinal detachment (SRD), and macular edema. **E** After treatment, fundus fluorescein angiography showing a hypofluorescent disc and a remarkable reduction of macular leakage. **F** Notice the reduction of retinal nerve fiber layer thickness and macular edema

**Fig. 2:**
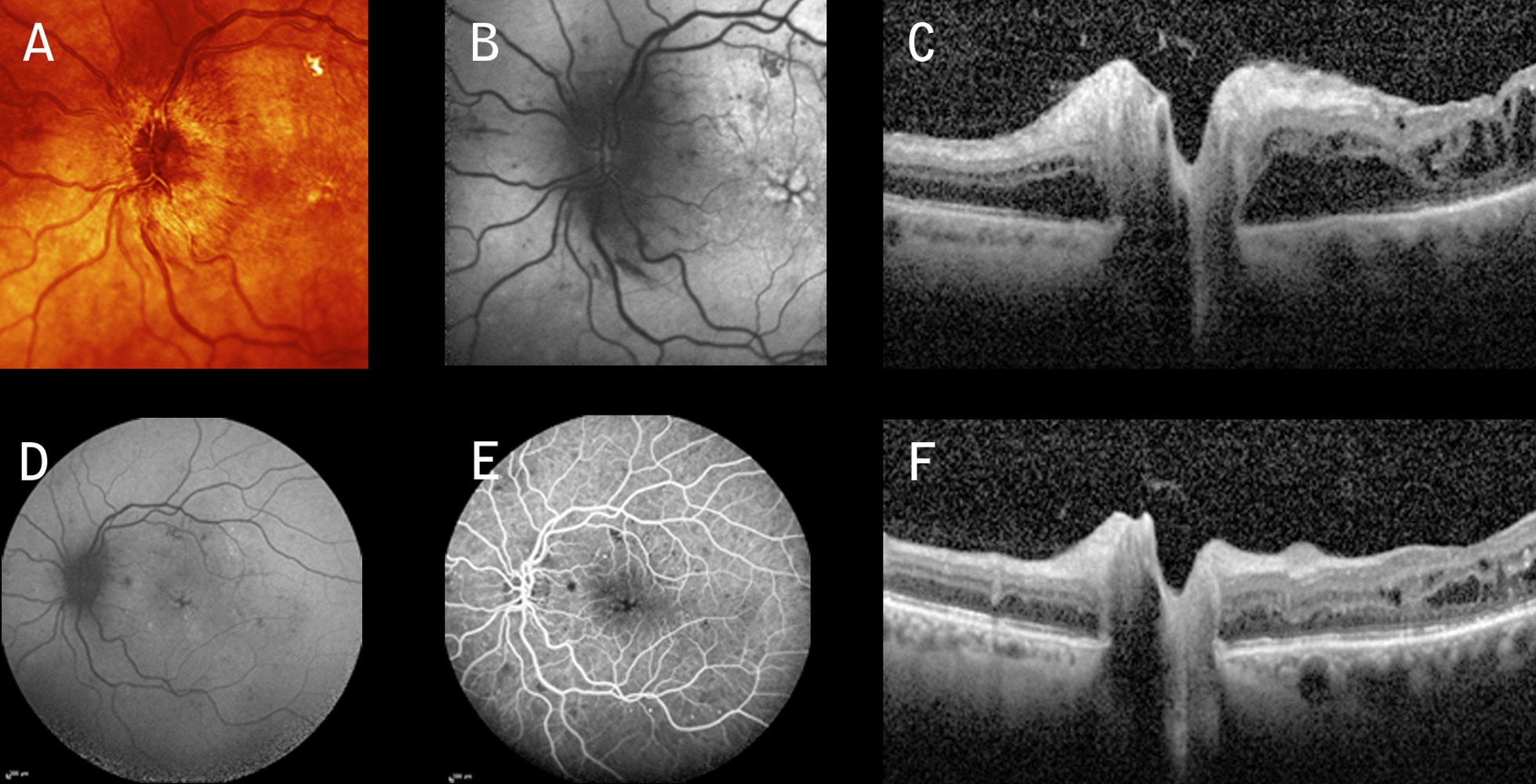
**A** Infrared imaging and **B** Fundus autofluorescence showing evidence of moderate-to-severe optic disc edema, peripapillary hypoautofluorescence, and foveal cysts in left eye. **C** Spectral domain optical coherent tomography showing marked edema of the optic disc and macular edema. Five weeks post-treatment **D** autofluorescence, **E** fundus fluorescein angiography, and **F** spectral domain optical coherent tomography showing marked regression of disc swelling with mild signs of macular edema, respectively

A 60-year-old Caucasian lady with poorly controlled type 2 diabetes mellitus (DM) presented herself 3 days after an acute drop in visual acuity in the right eye (OD), with a vision of 6/360. The patient was on oral hypoglycemic agents, and the last measured glycated hemoglobin (HbA1c) was 10.9%. Intraocular pressure (IOP) was 15 mmHg in both eyes (OU). The patient was phakic and had a normal anterior examination bilaterally, without signs of afferent pupillary defect (APD). Fundus examination and fundus autofluorescence (FAF) showed, respectively, a swollen optic disc and diffuse peripapillary hypoautofluorescence in OD (Fig. 1A, B) and a moderate nonproliferative diabetic retinopathy (NPDR) in OU. Fundus fluorescein angiography (FFA) highlighted dye leakage from the optic disc, while spectral domain optical coherent tomography (SD-OCT) imaging in OD revealed an abnormal increase in the retinal nerve fiber layer (RNFL) thickness and macular edema (ME) (Fig. 1B, C). Magnetic resonance imaging (MRI) scan of the brain and the laboratory work-up including inflammatory/infective etiologies and sedimentation rate (ESR) produced normal results. The patient was diagnosed with severe DP, and after informed consent she received an IAI (2 mg/0.05 mL) (Eylea Bayer Pharma AG) associated with a STTAI (40 mg/mL) (Kenacort Bristol-Myers Squibb Australia Pty Ltd). Seven weeks following the injections, the best corrected visual acuity (BCVA) in OD was 20/30 with an almost complete resolution of the optic disc edema (Fig. 1E–H). Within 30 days from the first presentation, the patient’s left eye (OS) exhibited more or less the same clinical picture as OD (Fig. 2A–C), with a BCVA of 6/60. The patient underwent the same therapy associated with a remarkable anatomic improvement (Fig. 1D–G) and a BCVA of 20/25 within 5 weeks. IOP values remained in the normal range at 6 months.

### Case 2

**Fig. 3:**
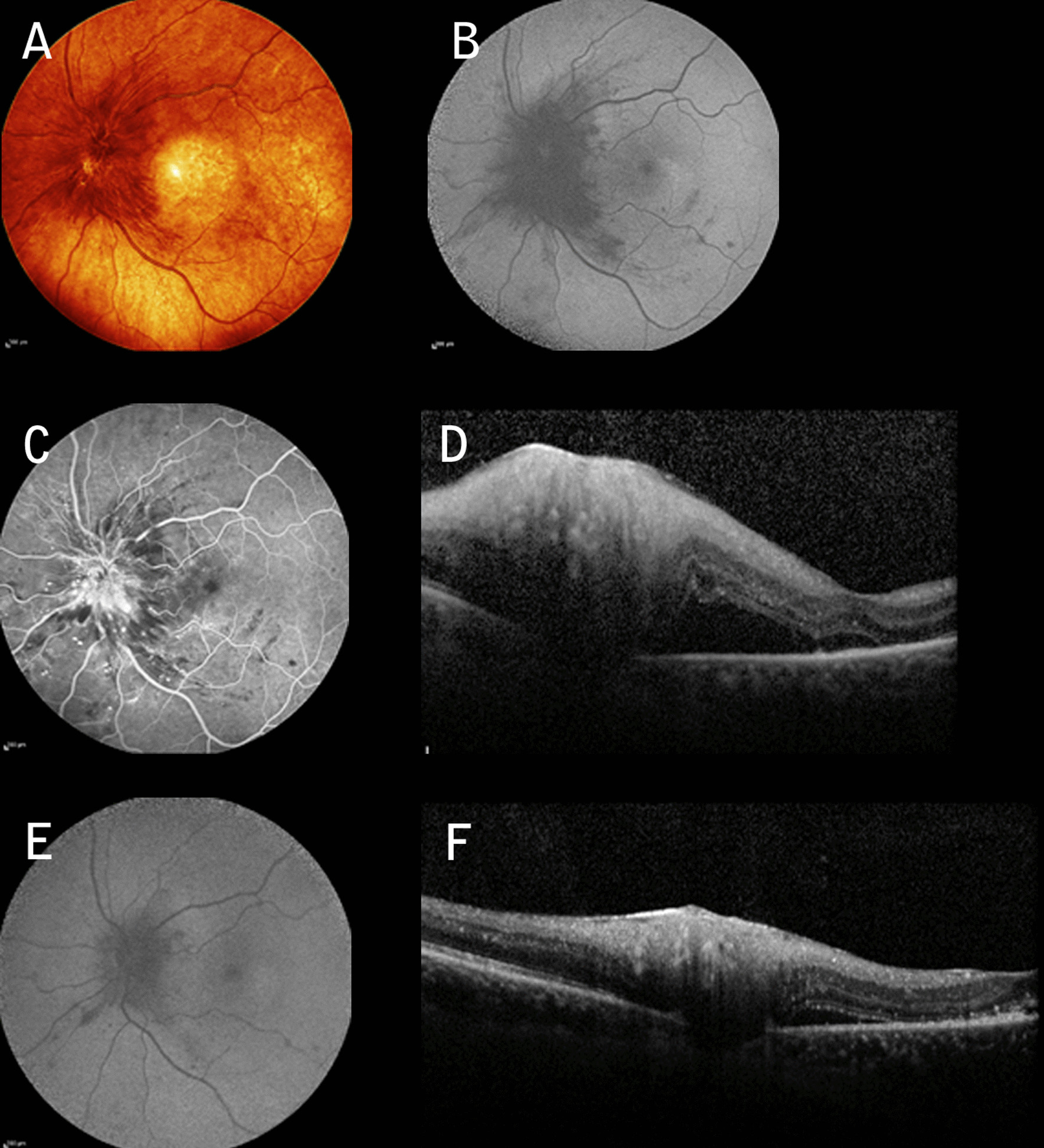
**A** Infrared imaging showing severe diabetic papillopathy with **B** diffuse hypoautofluorescence; **C** late venous phase associated with hyperfluorescent telangiectatic vessels associated with moderate leakage and mild nonproliferative diabetic retinopathy; **D** Spectral domain-optical coherent tomography revealing diffuse retinal nerve fiber layer thickening and serous macular detachment in left eye. Five weeks post injections there is strong evidence of improvement in multimodal imaging (**E**, **F**)

A 57-year-old Caucasian male with a 10-year history of type 2 DM presented with sudden visual loss in OD. The patient was on insulin therapy (HbA1c 11.2%). The patient was phakic, BCVA was 6/120, IOP was 17 mmHg, and there were no signs of APD. Fundoscopy revealed a swelling of the optic disc with telangiectatic vessels, along with mild NPDR (Fig. 3A, B). Moderate disc leakage and serous macular detachment were noted on FFA and OCT (Fig. 3C, D). A neurologic consultation with cranial MRI and lab findings showed no abnormality. Five weeks after receiving IAI and STTAI, the patient’s BCVA returned to 20/30, with regression of the disc edema (Fig. 3E–G). The patient developed moderate steroid-induced hypertension after 3 weeks and was treated with topical antiglaucomatous drugs. Written consent to publish case details was obtained from both patients.

## Discussion

Above we present the clinical findings of three eyes (two patients) consistent with DP, undergoing combined therapy with locoregional corticosteroids and anti-VEGFs. Although the differential diagnosis of DP includes a variety of entities such as infectious/inflammatory optic neuropathies, central retinal vein occlusion, idiopathic intracranial hypertension, and hypertensive retinopathy, its main differential remains non-arteritic anterior ischemic optic neuropathy (NA-AION). Our cases were associated with low BCVA due to the severity of DP and the concomitant ME, while the timing and level of visual recovery clearly distinguish DP from an eventual NA-AION diagnosis [2, 6, 7]. Moreover, our cases were associated with angiographic features not showing delays of filling in the prelaminar portion of the optic disc. In addition, no dysfunction of this structure was observed during follow-up. Recent studies report that DP patients have a shorter disease duration and are relatively younger compared with AION patients [8].

Even though the exact pathogenesis of DP remains a subject of controversy, different theories have been proposed to explain its occurrence. The dysmetabolic events seem to be leading to epi-/peripapillary microangiopathy with consequent leakage into and around the optic nerve head (ONH), while others believe that these ischemic and toxic optic nerve head changes might be related to a circulatory compromise in the deeper laminar vascular structures and axoplasmic flow disruption [9, 10]. The interplay between inflammatory cytokines and VEGF might serve as a rationale for the successful employment of corticosteroids and anti-VEGF agents in DP-related edema [11].

The anti-inflammatory effects, inhibitory effects on VEGF synthesis, and role in reducing vascular permeability of corticosteroids associated with the synergistic rapid decrease of VEGF provided by anti-VEGF agents achieved good efficacy in our study. Given the speculative and dichotomous mechanism of disc swelling in DP and owing to the severe drop in vision of our cases, we opted for a dual therapy. Our choice of the subtenon route for the triamcinolone acetonide (TA) injection was mainly influenced by the lesser extent of complications compared with the intravitreal route, and by the lens status. Intravitreal TA (IVTA) has limitations, including elevated IOP, cataract progression, pseudo-endophthalmitis, and infectious endophthalmitis [12–14], while STTAI is not free of potential complications such as accidental injection directly into the choroidal or retinal circulation, perforation of the ocular bulb, occlusion of the central retinal artery, and cataract [15]. Another reason was that, through this approach, the intravitreal therapy would be eventually focused on the superficial resolution of venous engorgement and stabilization of the hematoretinal barrier, while STTAI performed superonasally would target the deep nerve vasculature and the consequent decongestion. Indeed, there are reports supporting the employment of peri-/retrobulbar injections to attain sufficient corticosteroid concentrations around the optic nerve [16, 17].

Numerous papers regarding eventual therapies in DP include periocular/intravitreal corticosteroids, intravitreal anti-VEGFs, and laser photocoagulation. Mansour *et al.* reported on the beneficial effects of subtenon betamethasone injection in six eyes with DP and ME, highlighting the anti-edema and angiostatic effects of corticosteroids, as well as the good safety profile with only one eye requiring antiglaucomatous therapy. The authors underlined the importance of superonasal/temporal injections to reach the optic nerve and macular area, respectively [18]. IVTA injection alone was associated with rapid improvement in vision from a baseline BCVA of counting fingers at 1 m in a DP case [19]. The main suggestion was to reserve such a treatment for bilateral cases with severe drop in vision.

The actual literature provides numerous papers reporting on the efficacy of anti-VEGF agents while accentuating the role of VEGF in the vascular permeability of telangiectatic disc vessels and macular capillaries, given that ME is a common finding in more than half of DP patients [20–23]. The common denominator of these cases is represented by the mild-to-moderate grade of severity of DP, the baseline BCVA > 0.1, and the lack of macular edema. In the absence of precise markers of disease severity, the aforementioned criteria might eventually guide the therapeutic choice.

We were able to find only one paper dealing with a combined approach in the case of a patient affected by DP, where the authors administered intravenous bevacizumab and IVTA injection [24]. In this case, compared with ours, visual recovery was noticed relatively early, presumably owing to the mild clinical severity of DP. There are many reports of off-label use of intravitreal triamcinolone in cases of persistent and refractory diabetic macular edema. In the comparison between IVTA and STTAI, Choi *et al.* reported that both routes had similar effects on diabetic macular edema (DME), but that IVTA increased IOP after 3 months [25]. Ozdek *et al.* found a threefold increase in the number of eyes showing a significant increase in IOP (> 21 mmHg) that had been undergoing IVTA compared with STTAI [26]. Regarding the risk of cataract progression, patients treated with STTAI showed an evident safety profile [27, 28]. Kurt *et al.* recently reported similar anatomic and functional improvements after both IVTA and STTAI administrations in diabetic eyes [29]. Moreover, in this study, both routes of injection led to a significant constriction of retinal arteries and veins and, as a result, to the decline of vascular permeability and leakage [30]. The choice of TA delivery in the ocular compartment is subject to various opinions, and we agree that further research including larger samples is warranted to confirm our findings.

In conclusion, we affirm that DP is a challenging and rare diagnosis. As shown in our study, a specific treatment combining sequential IAI and STTAI for severe cases associated with macular edema would be beneficial in terms of a relatively fast and safe recovery.

## Data Availability

All data generated or analyzed during this study are included in this manuscript.

## Abbreviations

DP: Diabetic papillopathy
DR: Diabetic retinopathy
STTAI: Subtenon triamcinolone acetonide injection
IAI: Intravitreal aflibercept injection
DM: Diabetes mellitus
HbA1c: Glycated hemoglobin
IOP: Intraocular pressure
OD: Right eye
OU: Both eyes
APD: Afferent pupillary defect
FAF: Fundus autofluorescence
NPDR: Nonproliferative diabetic retinopathy
FFA: Fundus fluorescein angiography
SD-OCT: Spectral domain optical coherence tomography
RNFL: Retinal nerve fiber layer
MRI: Magnetic resonance imaging
ME: Macular edema
ESR: Sedimentation rate
BCVA: Best corrected visual acuity
OS: Left eye
NA-AION: Non-arteritic anterior ischemic optic neuropathy
OCTA: Optical coherence tomography angiography
IVTA: Intravitreal triamcinolone acetonide
